# Evaluating the Impact of the Saudi Model of Care ST-Elevation Myocardial Infarction (STEMI) Pathway on Interfacility Communication Between a PCI-Capable Center and Referring Hospitals in the Qassim Region, Saudi Arabia

**DOI:** 10.7759/cureus.101290

**Published:** 2026-01-11

**Authors:** Mohammad A Aldakheel, Yasser A Bhat, Fatmah Alribdi, Ahmed A Almeman, Mohammed A Alanazi, Abdulsalam Alfawzan, Suliman A Alradhi, Abdulrahman Almesned, Sultan A Alsultan, Albandary Alanazi, Annalyn V Camba, Query Ann D Rimando, Khuzama Alkhalaf, Musa M Alharbi, Abdullah Alqwaee

**Affiliations:** 1 Cardiology, Prince Sultan Cardiac Center, Buraidah, SAU; 2 Pediatric Cardiology, Prince Sultan Cardiac Center, Buraidah, SAU; 3 Saudi Model of Care, Qassim Health Cluster, Buraidah, SAU; 4 Pharmacology, Qassim University, Buraidah, SAU

**Keywords:** door-in-door-out (dido) time, door-to-balloon (d2b) time, interfacility communication, saudi arabia, stemi

## Abstract

Background: Timely reperfusion is critical in ST-elevation myocardial infarction (STEMI), especially when patients initially present to hospitals without percutaneous coronary intervention (PCI) capability. Door-to-balloon (D2B) time and door-in-door-out (DIDO) time are very vital for the clinical outcome of the STEMI patients. The STEMI Pathway, implemented under the Saudi Model of Care (SMoC), is a regional initiative to streamline interfacility communication and transfers between 16 hospitals (including one PCI-capable center) in the Qassim region of Saudi Arabia. Operated by Prince Sultan Cardiac Center Qassim (PSCCQ), the pathway is coordinated and supported by the Qassim Health Cluster as part of its ongoing efforts to enhance acute cardiac care services.

Aim: This study aims to assess the DIDO and D2B time for STEMI patients reported to PSCCQ in 2023 and 2024.

Methods: A retrospective study was conducted in March 2025 using data from STEMI cases transferred from non-PCI hospitals in Qassim province to the sole PCI center (PSCCQ) during 2023 and 2024. Important information recorded was arrival (door-in) at the receiving hospital, departure from the referring hospital (door-out), and balloon inflation time at the receiving hospital. From these, DIDO time (time of transfer out from a non-PCI facility to time of arrival at the referral hospital) and overall D2B time (from referral hospital arrival to PCI balloon inflation) were calculated.

Results: STEMI interfacility referrals increased from 312 in 2023 to 392 in 2024, indicating expanded utilization of the pathway. The DIDO time improved markedly from a median of 48 minutes in 2023 to 35 minutes in 2024. Likewise, median D2B decreased from 118 minutes in 2023 to 99 minutes in 2024. These improvements brought the median DIDO time closer to the recommended benchmark (≤30 minutes), while the median D2B time, which was already within the recommended ≤120 minutes in both 2023 and 2024, showed further improvement in 2024.

Conclusion: Implementation of the SMoC STEMI pathway was associated with significantly enhanced interfacility communication and expedited transfers. Specifically, DIDO improved significantly in 2024 and approached the international benchmark of ≤30 minutes. Median D2B times, already within the recommended ≤120 minutes in 2023, showed further improvement in 2024, directly corresponding to the improved DIDO times. These findings underscore the value of coordinated regional STEMI networks in optimizing time-sensitive cardiac care.

## Introduction

ST-elevation myocardial infarction (STEMI) is a life-threatening emergency condition requiring rapid restoration of coronary blood flow to minimize myocardial damage and reduce mortality [1]. Primary percutaneous coronary intervention (PCI) remains the preferred reperfusion strategy for STEMI management when performed promptly; however, timely access remains a challenge [2]. Achieving timely reperfusion is challenging when patients first present to non-PCI capable hospitals, as interfacility transfer results in additional delay [3]. Current American College of Cardiology/American Heart Association (ACC/AHA) guidelines (2025) recommend a first medical contact or initial hospital door-to-balloon (D2B) time of ≤90 minutes for patients directly presenting to a PCI-capable hospital, or ≤120 minutes for patients requiring transfer from a non-PCI-capable facility [4]. It is reported that >70% of STEMI patients require interhospital transfer for PCI, with many experiencing delays exceeding the recommended time (≤120 minutes) [5]. In such cases, the clinical guidelines recommend a door-in-door-out (DIDO) time, defined as the interval between arrival at a non-PCI hospital and departure for a PCI-capable center, which becomes a crucial determinant of total ischemic time [6]. Clinical guidelines recommend a DIDO time of ≤30 minutes; however, achieving this remains challenging, and prolonged delays are associated with worse clinical outcomes [3]. A meta-analysis reported that the mortality rate among STEMI patients with DIDO time ≤30 minutes was significantly lower (53.3% lower) compared to patients with DIDO >30 minutes [7].

Well-structured and organized STEMI pathways can help minimize interfacility transfer delays by improving communication and streamlining protocols. The existing literature has shown that implementing structured STEMI pathways can significantly reduce D2B time and improve treatment outcomes [8]. In the United States, achieving the recommended DIDO time below 30 minutes has proven challenging, as the data shows a median DIDO time of 53 minutes, and 44% of transfer patients are unable to achieve the recommended D2B time of ≤120 minutes, highlighting significant delays in interfacility coordination and timely reperfusion [9]. To address delays in STEMI care, best practices emphasize early ECG acquisition [10], prompt activation of the PCI team before arrival at a PCI-capable hospital [11], and streamlined ambulance transfer logistics [10]. Similarly, the use of modern communication tools, such as messaging applications or dedicated hotlines, between referral emergency departments, ambulance services, and the PCI-capable center can facilitate efficient STEMI care and improve outcomes. A regional STEMI network in North Cairo utilized WhatsApp to connect physicians across hospitals, enabling immediate ECG sharing and cath lab activation, leading to a significant reduction in D2B time and rapid revascularization via PCI [8]. Likewise, the “Code STEMI” protocol in Riyadh, which uses single-call catheterization laboratory activation, has substantially improved the timeliness of PCI delivery within the hospital [12].

Qassim region context

In the Qassim region of Saudi Arabia, Prince Sultan Cardiac Center, Qassim (PSCCQ) is the only PCI-capable center having a hub-and-spoke model for STEMI care serving multiple non-PCI peripheral hospitals. In PSCCQ, the coordinated regional pathway standardizes the diagnosis, stabilization, and transfer process through 24/7 specialist-led communication, utilizing mobile calls, a dedicated hotline, and encrypted WhatsApp® messaging. These mechanisms have significantly reduced interfacility delays and improved the timeliness of reperfusion therapy. Therefore, this study aims to evaluate the impact of the STEMI pathway by comparing key performance indicators, including DIDO and D2B times, before its implementation in 2023 and after in 2024.

## Materials and methods

Study design

A retrospective observational study was used to assess transfer times for STEMI patients in the Qassim region of Saudi Arabia. This study followed a single-arm pre-post design, comparing consecutive yearly cohorts (2023 vs. 2024). A parallel control group was not established, as withholding pathway-driven improvements from eligible STEMI patients would have been ethically unacceptable.

Setting

Data were collected from the STEMI Pathway program, the STEMI network in the Qassim region, covering 2023-2024 and comprising 16 participating hospitals: 15 referring hospitals without PCI capability and one hub hospital (PSCCQ) with 24/7 PCI services.

Inclusion and Exclusion Criteria

The study included all STEMI patients who presented to a referral hospital emergency department and were transferred to PSCCQ for primary PCI via the STEMI pathway. Patients who received fibrinolysis at the referral center or who were not transferred for any reason were excluded from the study.

Data Collection

For patients who met the above inclusion/exclusion criteria, the following time points were recorded in the STEMI Pathway registry: Door-in time: arrival/triage time at the referral hospital’s emergency room (ER); door-out time: time the patient physically left a non-PCI capable hospital ER en route to PSCCQ; first device activation time: time of first device activation in the cardiac catheterization laboratory at PSCCQ (defined in this study as the time of first balloon inflation during primary PCI).

Using these timestamps, two key performance intervals were calculated for each case: DIDO time (the interval from ED arrival at the PCI-capable hospital to departure from that non-PCI facility (door-out minus door-in), reflecting the speed and efficiency of the initial hospital in diagnosing the STEMI and preparing the patient for immediate transfer to a specialized center), and D2B time (the interval from arrival at the PSCCQ to the first balloon inflation encompasses the entire process). This includes the initial ECG diagnosis, activation, transportation to the cath lab, initiation of PCI, and the subsequent inflation of the balloon or placement of the stent. Therefore, it reflects the overall speed of treatment.

STEMI Pathway implementation

The STEMI Pathway was launched in late 2022 as a national initiative under the Saudi Model of Care (SMoC) within the Qassim Health Cluster to reduce treatment delays for STEMI patients. Its main components included standardized protocols for immediate ECG acquisition by triage nurses, interfacility communication via a secure WhatsApp group to transmit ECGs and activate the PCI center, and official transport coordination via the referring hospital ambulance, in collaboration with the Saudi Red Crescent. Workflow optimization was reinforced through structured checklists to enable parallel processes (IV access, laboratory tests, medications). Multidisciplinary training sessions for physicians, nurses, and ambulance staff were conducted, and weekly performance feedback reports were disseminated across referring hospitals to reinforce adherence and accountability.

Outcomes and benchmarks

The primary outcomes were the median DIDO time and the median D2B time for each year (2023 and 2024) to compare improvement. According to the guidelines, the target for DIDO was ≤30 minutes [3]; however, for D2B was ≤90 minutes for patients being transferred as recommended by a meta-analysis [4]. Additionally, the secondary outcome was to evaluate the total number of STEMI patients transferred each year to assess the network utilization.

Patients’ confidentiality and access to data

Patient confidentiality was strictly maintained throughout the study. As part of the quality improvement review of the STEMI Pathway initiative, data from the respective years were assessed, with all participating hospitals providing data through the Qassim Health Cluster’s Cardiology service line, thereby protecting patient privacy. This study was approved by the Qassim Health Cluster Research Ethics Committee; no informed consent was required as only de-identified registry data were used.

Statistical analysis

Descriptive statistics were used to summarize the transfer times in 2023 and 2024. Medians of DIDO and D2B times were compared between the two years. To quantify improvement, between 2023 and 2024, for DIDO and D2B, the percentage change was calculated. Data analysis was performed using IBM SPSS Statistics for Windows, Version 24 (Released 2016; IBM Corp., Armonk, New York, United States). Continuous variables were reported as both mean ± standard deviation (SD) and median with interquartile range (IQR). Between-group comparisons were tested using t-tests or Mann-Whitney U tests, while categorical variables (e.g., gender, comorbidities) were analyzed with chi-square tests. A p-value <0.05 was considered statistically significant.

## Results

STEMI transfer volume

A total of N=312 (100%) STEMI patients were transferred from referral hospitals to PSCCQ in 2023 under the STEMI Pathway; in 2024, this increased by 25.6% to N=392 (100%).

Baseline characteristics

Baseline demographic and clinical characteristics of STEMI patients in 2023 and 2024 are summarized in Table 1.

**Table 1:** Key performance indicators (KPIs) for STEMI transfers to PSCCQ, 2023 vs. 2024. STEMI: ST-elevation myocardial infarction; PSCCQ: Prince Sultan Cardiac Center Qassim; PCI: percutaneous coronary intervention

Metric	2023 (N=312)	2024 (N=392)	Absolute Improvement
Door-in-door-out (DIDO) time at referral hospital (median, minutes)	48	35	13 minutes (-27%)
Door-to-balloon time (D2B) (median, minutes) from referral door-in to PCI balloon inflation	118	99	19 minutes (-16.1%)

Random sampling and adjustments confirmed that the two cohorts were largely comparable with respect to age, sex, nationality, and comorbidity profiles. This indicates that the observed improvement in D2B times in 2024 is unlikely to be explained by differences in patient case-mix but rather reflects the impact of enhanced interfacility communication and the implementation of the STEMI Pathway.

Transfer time intervals

Key performance metrics for 2023 and 2024 are summarized in Table 2.

**Table 2:** Baseline demographic and clinical characteristics of STEMI patients (2023 vs. 2024). CAD: coronary artery disease; MI: myocardial infarction; STEMI: ST-elevation myocardial infarction

Characteristic	2023, N=312 (100%)	2024, N=392 (100%)	P-value
Mean age (years), SD	55.18 (12.26)	54.87 (12.11)	0.720
Male sex, n (%)	249 (83)	349 (89)	0.045
Saudi nationality, n (%)	240 (77)	278 (71)	0.143
Diabetes mellitus, n (%)	165 (53)	188 (48)	0.350
Hypertension, n (%)	143 (46)	164 (42)	0.420
Dyslipidemia, n (%)	84 (27)	121 (31)	0.380
Current/former smokers, n (%)	56 (18)	113 (29)	0.021
Prior CAD/MI/revascularization, n (%)	43 (14)	31 (8)	0.110

In 2023, the median DIDO time was 48 minutes (IQR 41-73), whereas in 2024 it decreased to 35 minutes (IQR 32-38), representing a 13-minute (27%) improvement. Comparative analysis confirmed that this reduction was statistically significant (p = 0.004) (Table 3).

**Table 3:** Comparative analysis of process measures (2023 vs. 2024). DIDO: door-in-door-out; D2B: door-to-balloon; NS: not significant

Variable	Mean 2023	Mean 2024	Difference (95% CI)	P-value
Door-to-ECG (min)	3.53	3.43	0.10 (-0.47, 0.67)	0.732 (NS)
DIDO (min)	111	48	62.4 (20.2, 104.6)	0.004
D2B (min)	111.3	91.1	20.2 (15.4, 25.1)	<0.001

By contrast, door-to-ECG time remained stable between the two years, with a median of two minutes in both cohorts (p = 0.73), indicating that early diagnostic performance was already optimized before pathway implementation (Tables 2, 3).

Subgroup analysis by referral hospital

When analyzed individually, nearly all referring centers showed consistent reductions in both DIDO and D2B times between 2023 and 2024. Improvements were most pronounced in high-volume centers such as Buraydah Central and King Saud Hospital in Unaizah, though smaller hospitals showed similar directional trends. Door-to-ECG times exhibited minimal change, reinforcing that diagnostic efficiency was already strong at baseline. Some low-volume hospitals had insufficient data for reliable estimates; these are indicated with an asterisk (*) in Tables 4-5.

**Table 4:** Subgroup analysis of process measures by referring hospital (2023). Values are presented as median (IQR) and mean ± SD. * indicates insufficient data for reliable estimates. DIDO: door-in-door-out; D2B: door-to-balloon

Variable	Referred From the Hospital	Mean	SE Mean	StDev	Q1	Median	Q3
Door-to-ECG_Minutes	Al-Asyah Hospital	4.50	1.13	3.57	1.00	3.00	9.00
	Al-Badaa General Hospital	4.71	0.99	4.09	1.50	3.00	6.50
	Al-Bikeriah General Hospital	4.23	0.54	2.78	2.00	4.00	6.00
	Al-Rass General Hospital	2.85	0.41	2.59	1.00	2.00	4.50
	Ouyoon Aljowaa Hospital	4.36	0.78	2.90	2.00	3.50	7.25
	Al-Mothneb General Hospital	4.89	0.76	3.22	2.00	4.50	8.00
	Riyad Alkhobaraa General Hospital	4.20	0.76	2.93	1.00	5.00	6.00
	Buraydah Central Hospital	3.13	0.42	3.31	1.00	2.00	5.00
	Qassim National Hospital	4.67	2.03	3.51	1.00	5.00	8.00
	Qusaybaa General Hospital	5.00	0.00	0.00	*	5.00	*
	King Saud Hospital in Unaizah	3.14	0.38	2.92	1.00	2.00	5.00
	Al-Habib Medical Group	4.67	0.84	2.07	2.75	5.00	5.75
	Hayat National Hospital	6.00	*			6.00	*
	Guwarah General Hospital	1.00	0.00	0.00	*	1.00	*
DIDO_Minutes	Al-Asyah Hospital	37.9	5.08	16.06	30.5	35.0	40.0
	Al-Badaa General Hospital	37.7	1.81	7.46	33.0	36.0	38.5
	Al-Bikeriah General Hospital	38.3	1.67	8.52	34.8	37.0	38.3
	Al-Rass General Hospital	34.8	0.88	5.66	31.0	35.0	38.0
	Ouyoon Aljowaa Hospital	36.9	1.90	7.10	33.0	35.5	41.0
	Al-Mothneb General Hospital	41.6	2.78	11.8	34.8	37.0	51.0
	Riyad Alkhobaraa General Hospital	40.5	2.38	9.21	35.0	38.0	39.0
	Buraydah Central Hospital	56.9	18.2	144.2	31.0	35.0	39.0
	Qassim National Hospital	*					
	Qusaybaa General Hospital	39.0	1.00	1.41	*	39.0	*
	King Saud Hospital in Unaizah	65.2	22.0	169.1	31.0	34.0	38.0
	Al-Habib Medical Group	*					
	Hayat National Hospital	*					
	Guwarah General Hospital	33.5	1.50	2.12	*	33.5	*
D2B_Minutes	Al-Asyah Hospital	106.0	2.73	8.64	101.0	104.0	105.3
	Al-Badaa General Hospital	102.7	4.05	16.7	99.5	103.0	106.5
	Al-Bikeriah General Hospital	100.3	1.36	6.92	94.8	102.0	106.0
	Al-Rass General Hospital	106.9	1.23	7.87	103.0	105.0	109.0
	Ouyoon Aljowaa Hospital	100.7	2.33	8.71	97.5	103.0	107.0
	Al-Mothneb General Hospital	113.3	3.89	16.5	104.8	107.0	115.8
	Riyad Alkhobaraa General Hospital	112.9	4.04	15.7	105.0	109.0	111.0
	Buraydah Central Hospital	95.8	1.82	14.4	86.0	97.0	106.0
	Qassim National Hospital	103.7	3.71	6.43	99.0	101.0	111.0
	Qusaybaa General Hospital	99.0	1.00	1.41	*	99.0	*
	King Saud Hospital in Unaizah	103.4	1.74	13.4	99.0	104.0	107.0
	Al-Habib Medical Group	95.3	9.06	22.2	78.0	91.0	106.5
	Hayat National Hospital	115.0	*			115.0	*
	Guwarah General Hospital	96.0	2.00	2.83	*	96.0	*

**Table 5:** Subgroup analysis of process measures by referring hospitals (2024). Values are presented as median (IQR) and mean ± SD. * indicates insufficient data for reliable estimates. DIDO: door-in-door-out; D2B: door-to-balloon

Variable	Referred From the Hospital	Mean	SE Mean	StDev	Q1	Median	Q3
Door-to-ECG_Minutes	Al-Asyah Hospital	4.50	1.13	3.57	1.00	3.00	9.00
	Al-Badaa General Hospital	4.71	0.99	4.09	1.50	3.00	6.50
	Al-Bikeriah General Hospital	4.23	0.54	2.78	2.00	4.00	6.00
	Al-Rass General Hospital	2.85	0.41	2.59	1.00	2.00	4.50
	Ouyoon Aljowaa Hospital	4.36	0.78	2.90	2.00	3.50	7.25
	Al-Mothneb General Hospital	4.89	0.76	3.22	2.00	4.50	8.00
	Riyad Alkhobaraa General Hospital	4.20	0.76	2.93	1.00	5.00	6.00
	Buraydah Central Hospital	3.13	0.42	3.31	1.00	2.00	5.00
	Qassim National Hospital	4.67	2.03	3.51	1.00	5.00	8.00
	Qusaybaa General Hospital	5.00	0.00	0.00	*	5.00	*
	King Saud Hospital in Unaizah	3.14	0.38	2.92	1.00	2.00	5.00
	Al-Habib Medical Group	4.67	0.84	2.07	2.75	5.00	5.75
	Hayat National Hospital	6.00	*			6.00	*
	Guwarah General Hospital	1.00	0.00	0.00	*	1.00	*
DIDO_Minutes	Al-Asyah Hospital	37.9	5.08	16.06	30.5	35.0	40.0
	Al-Badaa General Hospital	37.7	1.81	7.46	33.0	36.0	38.5
	Al-Bikeriah General Hospital	38.3	1.67	8.52	34.8	37.0	38.3
	Al-Rass General Hospital	34.8	0.88	5.66	31.0	35.0	38.0
	Ouyoon Aljowaa Hospital	36.9	1.90	7.10	33.0	35.5	41.0
	Al-Mothneb General Hospital	41.6	2.78	11.8	34.8	37.0	51.0
	Riyad Alkhobaraa General Hospital	40.5	2.38	9.21	35.0	38.0	39.0
	Buraydah Central Hospital	56.9	18.2	144.2	31.0	35.0	39.0
	Qassim National Hospital	*					
	Qusaybaa General Hospital	39.0	1.00	1.41	*	39.0	*
	King Saud Hospital in Unaizah	65.2	22.0	169.1	31.0	34.0	38.0
	Al-Habib Medical Group	*					
	Hayat National Hospital	*					
	Guwarah General Hospital	33.5	1.50	2.12	*	33.5	*
D2B_Minutes	Al-Asyah Hospital	106.0	2.73	8.64	101.0	104.0	105.3
	Al-Badaa General Hospital	102.7	4.05	16.7	99.5	103.0	106.5
	Al-Bikeriah General Hospital	100.3	1.36	6.92	94.8	102.0	106.0
	Al-Rass General Hospital	106.9	1.23	7.87	103.0	105.0	109.0
	Ouyoon Aljowaa Hospital	100.7	2.33	8.71	97.5	103.0	107.0
	Al-Mothneb General Hospital	113.3	3.89	16.5	104.8	107.0	115.8
	Riyad Alkhobaraa General Hospital	112.9	4.04	15.7	105.0	109.0	111.0
	Buraydah Central Hospital	95.8	1.82	14.4	86.0	97.0	106.0
	Qassim National Hospital	103.7	3.71	6.43	99.0	101.0	111.0
	Qusaybaa General Hospital	99.0	1.00	1.41	*	99.0	*
	King Saud Hospital in Unaizah	103.4	1.74	13.4	99.0	104.0	107.0
	Al-Habib Medical Group	95.3	9.06	22.2	78.0	91.0	106.5
	Hayat National Hospital	115.0	*			115.0	*
	Guwarah General Hospital	96.0	2.00	2.83	*	96.0	*

## Discussion

STEMI requires rapid treatment to limit infarct size and improve survival. PCI is the standard, with the 2025 ACC/AHA guidelines recommending a D2B time of ≤90 minutes for PCI-capable hospitals and ≤120 minutes for referral patients to PCI balloon inflation [4]. Delays, particularly in DIDO time at non-PCI hospitals, affect outcomes. A DIDO ≤30 minutes is associated with lower mortality, underscoring the need for efficient regional STEMI networks [3]. Streamlined pathways, including rapid ECG acquisition, early PCI activation, and modern communication tools, have shown promise in improving transfer times and outcomes.

In summary, the baseline characteristics of STEMI patients in 2023 and 2024 were remarkably consistent across age, gender, nationality, and comorbidities (Table 5). This similarity reduces the likelihood that the observed improvements were attributable to patient differences, supporting the interpretation that the STEMI Pathway itself was the primary driver of faster treatment times.

Comparison with other regions

Our study findings illustrate the positive impact of a structured regional STEMI pathway on interfacility transfer efficiency in the Qassim region. Compared with 2023, we observed a decrease in the DIDO time STEMI patients spent at referring hospitals (13 minutes shorter) and a corresponding reduction in overall D2B time by 19 minutes. These improvements are primarily attributable to the implementation of the structured STEMI Pathway under the SMoC, which standardized communication, triage, and transfer processes across the Qassim Health Cluster. These findings are clinically significant, and our analysis of 2024 data shows that patients were treated within the critical 120-minute window recommended for STEMI transfer [13,14]. Another study conducted in the Kingdom of Saudi Arabia at King Faisal Specialist Hospital and Research Centre in Riyadh achieved and sustained an international benchmark for D2B time of less than 90 minutes through effective multidisciplinary collaboration [15]. Another study in the North Cairo STEMI network, which employed direct communication via WhatsApp, saw about a 10-minute reduction in D2B time and significant improvements in care delivery [8]. In other studies, such as those from the USA, the median D2B time was 63 minutes [16], whereas in European studies it was 60 minutes [17]. By comparison, the STEMI Pathway in 2024 would likely have a significantly higher percentage of patients meeting the 120-minute goal (given a median of 99, one can infer a substantial majority under threshold). This suggests that Qassim’s regional model, despite the challenges of distance and a single PCI center, has achieved performance comparable to or exceeding that of larger systems in more developed settings. Achieving an average DIDO of 35 minutes is particularly commendable; although it remains above the ideal 30 minutes, it represents a significant step toward optimal care. Thus, STEMI Pathway’s results demonstrate that targeted regional initiatives can markedly close the gap toward this goal.

Similarly, the DIDO time improved by 13 minutes, having a 27% improvement from 2023 to 2024. However, it still needed to be further enhanced to achieve the ideal time as per the guidelines of ≤30 minutes [18]. Achieving the guideline-set ideal time for DIDO is crucial; however, our findings showed an improvement from 48 minutes in 2023 to 35 minutes in 2024. Other studies reported higher DIDO times than our findings; for example, in Portugal, the median DIDO time was 82 minutes, and only 1.3% of patients were transferred in 30 minutes or less [19]. Similarly, in another Canadian study, the median DIDO time was 55 minutes [20]. A study in the same Gulf region, conducted in Bahrain, the DIDO standard time goal was achieved in 45% of the patients with a median time of 32.5 minutes [21]. Overall, different factors are contributing to this delay in attaining the targeted DIDO times at the referring hospital, including awaiting transport and emergency department delays, diagnostic dilemmas, and non-diagnostic initial electrocardiography (ECG; median 81 minutes; IQR 64-110.5 minutes) [22], and difficulties interpreting the ECG [23]. Moreover, the increase in primary PCI cases in Qassim (392 in 2024, up from 312 in 2023) parallels the Cairo experience, where implementing a network led to a rise in primary PCI rates (from ~60% to 77% of STEMI cases) and a corresponding drop in use of fibrinolysis [8]. Although our data did not explicitly track thrombolytic use, the higher transfer volume suggests that more patients are now managed with primary PCI as the default strategy, consistent with guideline recommendations when timely PCI is available. This likely has downstream benefits for patient outcomes, as primary PCI is generally more effective than fibrinolysis in capable systems.

Interfacility communication and process changes

The substantial reduction in DIDO time suggests that referring hospitals were able to initiate the transfer process more quickly under the refined STEMI Pathway protocol. Several factors contributed to this improvement:

Streamlined Communication

A key element of the STEMI Pathway is prompt communication between the referral ER and PSCCQ. In practice, this meant earlier activation of PSCCQ’s catheterization lab team (often via a single-call system) once STEMI was confirmed at the referring hospital. By 2024, refined STEMI Pathway protocols ensured real-time communication, with no delay in decision-making, and prepared the PCI center in advance [8]. Our findings align with experiences elsewhere; for instance, the introduction of a direct paging/activation system in Riyadh’s post-code-STEMI program greatly reduced D2B therapy, with a median of 76.5 minutes, compared with a median of 107 minutes in pre-code-STEMI patients, increasing the proportion of patients treated within target times [12]. Another study in Taiwan reported that Acute Myocardial Infarction Software Aids resulted in a significant reduction in mean D2B time from 76.6 minutes to 66.7 minutes [24].

Faster Triage and Diagnosis

The reduction in DIDO also reflects quicker ER processes at referral hospitals. By 2024, ER staff were likely more adept at rapid triage and obtaining an ECG within the first minutes of arrival (guidelines advise ECG within 10 minutes of presentation) [3,25]. System improvement in the ER, like reducing the door-to-ECG time, streamlining STEMI detection and cardiology team activation time, reducing transfer time to the cath lab, and faster STEMI diagnosis, are among the strategies to improve outcomes [26]. Similarly, in our study, with STEMI Pathway training, emergency physicians and nurses can identify STEMI and trigger the pathway promptly. We did not directly measure door-to-ECG time in this report; however, an efficient door-to-ECG time (ideally ≤10 minutes) is a precursor to reducing door-in-to-door-out time. It is plausible that STEMI Pathway initiatives reinforced the importance of immediate ECG for patients with chest pain and perhaps provided feedback on each hospital’s performance, thereby facilitating faster diagnostic workflows.

Reduced On-Site Treatment Delays

In some cases, initial management, such as administering dual antiplatelet therapy (aspirin and P2Y12 inhibitors [27]) or unfractionated heparin [28,29], influences decisions regarding the optimal timing of transfer and treatment [30].

Emergency Medical Services (EMS) Coordination

Enhanced coordination with EMS (ambulance teams) likely also contributed to shorter DIDO [31,32]. In a well-functioning STEMI network, the referring hospital notifies EMS as soon as the decision to transfer is made, ensuring an ambulance is either already on site or en route during the patient’s brief evaluation. Another study in the Kingdom of Saudi Arabia reported a substantial decrease in average ambulance response times for interfacility transfers from 17 minutes to nine minutes due to the adoption of EMS smart solutions and improved coordination between referral hospitals and receiving centers [33]. The Saudi Heart Association's clinical guidelines emphasize the importance of strengthening local EMS services to meet recognized standards for acute coronary syndrome and facilitate timely transfers to appropriate facilities [34]. By 2024, pre-arranged protocols with Qassim Health Cluster’s Ambulance services ensured rapid dispatch for STEMI transfers. This contrasts with potential delays in 2023 when ambulances might have been called later in the process. Efficient EMS response is an important factor; patients who arrived by EMS with STEMI notification had shorter D2B times [32,35]. In Qassim, geographic distances are fixed, but improved coordination can reduce transport wait times.

Clinical implications

The reduction in treatment times observed is likely to have a meaningful clinical impact. While our study did not directly measure patient outcomes; however, the relationship between faster reperfusion and improved clinical outcomes is well-established for decades [36,37]. Shorter D2B times are associated with lower mortality, less infarct size, and better left ventricular function [8]. In our context, by cutting DIDO time by over a quarter, the STEMI Pathway could plausibly improve survival and reduce complications for STEMI patients. Longer transfer times used to be associated with higher mortality, shock, and heart failure among patients undergoing inter-hospital transfer [38]. In North Cairo, the in-hospital mortality dropped from 6.4% to 2.8% after implementing its fast-transfer network [8]. In another study in Indonesia, following implementation of the STEMI network, in-hospital mortality decreased from 9.6% to 7.1% [39]. We would expect the STEMI Pathway to confer similar benefits as more patients receive timely PCI. An integrated and well-organized STEMI network facilitates the selection of the optimal acute reperfusion strategy according to the individual clinical scenario [40].

Challenges and remaining gaps

Despite the progress, some patients in 2024 still experienced DIDO times above 30 minutes and D2B above 120 minutes. The ongoing challenge is achieving a DIDO time of ≤30 minutes in every patient with STEMI. Delays may be caused by clinical presentations that raise uncertainty regarding the diagnosis, and referrals from institutions that are geographically far enough away, even with a timely transfer protocol beginning. To address these, continuous quality improvement is needed. Strategies could involve regular feedback to each hospital on their DIDO performance [41], EMS services needed to be provided with patients care report and estimated time of arrival which is crucial for review and quality improvement purpose [42], training and simulation skills also play a vital role enhancing communication as well as collaboration [43], and STEMI diagnosis via ECG should be obtained within 10 minutes though first medical contact [44]. In fact, if paramedics in Qassim can identify STEMI en route to any hospital, it may become feasible in some cases to bypass the non-PCI capable facility and go directly to PSCCQ, further reducing total ischemic time (a strategy known to save time in other regions by ER bypass).

Another consideration is the increased volume of cases at the PCI center (392 in 2024, up from 312). While so far, the data suggest PSCCQ has managed the volume without slowing D2B on their end. Keeping in view the increasing cases in 2024 as compared to 2023, the rise in the number of cases can overburden the referral network and may exert further pressure, leading to delayed DIDO and D2B therapy from the referrals. In order to cope with this, the first medical contact needed to be enhanced further along with the STEMI network. To maintain performance and quality of services, it will be crucial to make sure that the cath lab team at PSCCQ has enough employees and that parallel processing is managed by many STEMI activations consecutively in an effective way. Interfacility communication also extends to post-PCI care; the STEMI Pathway has to coordinate returning patients to their referring hospitals or managing beds at the PSCCQ after PCI. A study based on QAPAS at PSCCQ in 2020 found that immediately returning post-PCI patients to the referring hospital can be safe in a way that these patients were having no major complication after PCI and were hemodynamically stable, returning the post-PCI patients to the referral hospitals can free up the bed capacity and cath lab staff to be ready for any new emergencies [45].

Limitations

This study is retrospective in nature, which introduces inherent limitations. First, it only compares DIDO and D2B times between 2023 and 2024, without identifying the specific factors responsible for the observed improvements. Second, clinical outcome data, such as mortality, infarct size, and heart failure incidence, were unavailable, limiting the ability to link faster reperfusion to improved prognosis directly. Third, the analysis was limited to a single PCI-capable hub in the Qassim region, which may limit the generalizability of the findings. Nevertheless, this study contributes to the growing body of evidence on the value of regional STEMI systems of care. The STEMI Pathway, implemented under the SMoC, demonstrates how structured protocols, clear communication, and continuous monitoring can significantly improve interfacility transfer efficiency.

Finally, while several Saudi studies have reported D2B times at PCI-capable centers, to the best of our knowledge, no prior national or regional report has described DIDO or transfer metrics. This limits direct benchmarking but underscores the novelty of our findings.

## Conclusions

The optimization of the STEMI pathway, implemented under the SMoC, has significantly improved interfacility communication and expedited care for STEMI patients in the Qassim region. This enhancement is reflected in the reduction of both DIDO and D2B times in 2024 compared to 2023. The establishment of a structured regional STEMI pathway has greatly streamlined interfacility transfer processes and ensured alignment with international benchmarks. As a result, the quality and timeliness of care for patients with STEMI in the Qassim region have markedly improved.
